# Risk-weighted apoB: a novel summary metric outperforming traditional lipid biomarkers in predicting coronary heart disease

**DOI:** 10.1093/eurheartj/ehaf1124

**Published:** 2026-01-22

**Authors:** Michaela B Rehman, Elias Björnson, Martin Adiels, Jakub Morze, Göran Bergström, Anders Gummesson, David Erlinge, Tove Fall, Ljubica Matic, Stefan Söderberg, Carl Johan Östgren, Chris J Packard, Jan Borén

**Affiliations:** Cardiology Department, Ramsay Santé, Médipôle Lyon-Villeurbanne, Villeurbanne, France; Institute of Medicine, University of Gothenburg, Gothenburg, Sweden; Institute of Medicine, University of Gothenburg, Gothenburg, Sweden; Institute of Medicine, University of Gothenburg, Gothenburg, Sweden; SciLifeLab, Department of Life Sciences, Chalmers University of Technology, Gothenburg, Sweden; Institute of Medicine, University of Gothenburg, Gothenburg, Sweden; Institute of Medicine, University of Gothenburg, Gothenburg, Sweden; Department of Clinical Genetics and Genomics, Region Västra Götaland, Sahlgrenska University Hospital, Gothenburg, Sweden; Department of Cardiology, Clinical Sciences, Lund University, Lund, Sweden; Department of Medical Sciences, Molecular Epidemiology, Uppsala University, Uppsala, Sweden; Department of Molecular Medicine and Surgery, Karolinska Institute, Stockholm, Sweden; Department of Public Health and Clinical Medicine, Umeå University, Umeå, Sweden; Center for Medical Image Science and Visualization (CMIV), Linköping University, Linköping, Sweden; Department of Health, Medicine and Caring Sciences, Linköping University, Linköping, Sweden; Institute of Cardiovascular and Medical Sciences, University of Glasgow, Glasgow, UK; Institute of Medicine, University of Gothenburg, Gothenburg, Sweden

**Keywords:** Mendelian randomization, Cardiovascular disease, apoB, Lp(a), LDL, LDL cholesterol, UK Biobank

## Abstract

**Background and Aims:**

LDL-C and non-HDL-C do not fully capture coronary heart disease (CHD) risk attributed to all apoB-containing lipoproteins. Use of apolipoprotein B (apoB) as a marker of total atherogenic particle number improves risk prediction, but risk may still be underestimated when triglyceride-rich lipoproteins (TRL/remnants) and lipoprotein(a) [Lp(a)] are elevated. The aim was to formulate a new metric—risk-weighted apoB (RW-apoB)—designed to capture risk from LDL, TRL/remnants, and Lp(a) in a single number.

**Methods:**

Based on previously published estimates of the relative atherogenicity of LDL, TRL/remnant, and Lp(a) particles, RW-apoB was developed (using UK Biobank data) as an atherogenicity-weighted apoB-sum calculated as: RW-apoB = 11.65×TG(mmol/L) + 0.215×lipoprotein(a)(nmol/L) + 0.736×apoB(mg/dL).

**Results:**

Assigning RW-apoB to individuals substantially reclassified their risk status. Compared with ranking by measured apoB, 52% of individuals were up- or down-ranked by ≥10 percentiles. About one-third of those in the top RW-apoB quintile—with elevated TRL and Lp(a) and a CHD event rate of 5.4%—were misclassified as lower risk by apoB. Conversely, individuals in the top measured apoB quintile but with low TRL and Lp(a) had a lower event rate (3.9%) and were correctly down-ranked. RW-apoB improved risk prediction, significantly increasing Harrell’s C-index relative to apoB (*P* < .0001). In statin-treated subjects, RW-apoB was potentially a better index of residual risk. RW-apoB consistently outperformed apoB as a risk predictor in Cox models across the UK Biobank and three other large population cohorts.

**Conclusions:**

RW-apoB represents not only particle number but also accounts for the higher atherogenicity of TRL and Lp(a). It offers clinically meaningful improvements in CHD risk stratification.

## Structured Graphical Abstract

**Graphical Abstract:**
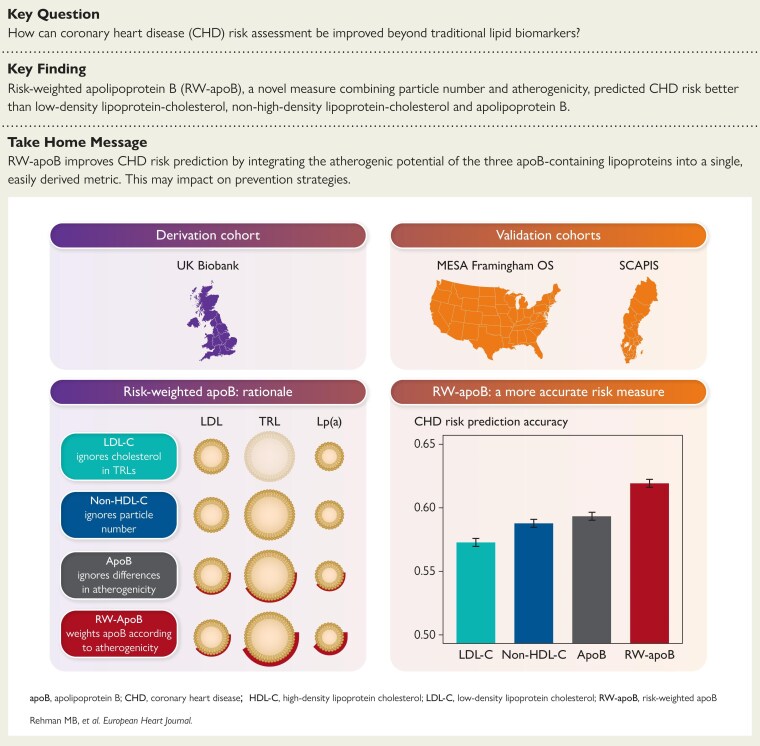
Risk-weighted apolipoprotein B (RW-apoB) provides a single, easily derived metric that integrates the number and atherogenicpotential of LDL, TRL and Lp(a). This metric more accurately reflects total atherogenic burden and coronary heart disease risk than LDLcholesterol,non-HDL-cholesterol or apoB alone. It substantially improves risk stratification and the reclassification of high-risk individuals, aswell as the identification of residual risk, across multiple large population-based cohorts.

**See the editorial comment for this article ‘Risk-weighted apolipoprotein B: is it worth the weight?’, by R.A. Hegele, https://doi.org/10.1093/eurheartj/ehag134.**

Translational Perspective This study reinforces the concept that the three apoB-containing lipoproteins—LDLs, triglyceride-rich lipoproteins, and lipoprotein(a)—have varying atherogenic potential. Risk-weighted apoB (RW-apoB) integrates the number and atherogenic potential of these atherogenic lipoproteins, offering superior CHD risk discrimination over LDL-C, non-HDL-C, or apoB alone. Cell culture studies and experiments in animal models and tissue explants of normal and diseased arteries are required to elucidate the physicochemical basis of the enhanced atherogenicity of triglyceride-rich lipoproteins and Lp(a) relative to LDL.

## Introduction

Apolipoprotein B (apoB)-containing lipoproteins play a central causal role in atherosclerosis. LDL is the most abundant lipoprotein class in the bloodstream and is established as a primary target for intervention in treatment guidelines.^1,2^ A range of effective pharmaceutical agents are available that permit a very low plasma concentration of LDL to be achieved routinely. However, it is now evident from clinical trials that even individuals with well-controlled LDL levels carry a substantial residual risk, a significant component of which can be attributed to other apoB-containing lipoproteins, namely triglyceride-rich lipoproteins (TRL) and their remnants, and lipoprotein(a) (Lp(a)).^3–8^ Recent genetic and observational studies have revealed that these lipoprotein classes exhibit greater atherogenicity per-particle than LDL. The increment in coronary heart disease (CHD) risk due to TRL/remnants has been estimated to be four to five times that of LDL on a per-particle basis.^9,10^ Likewise, per-particle Lp(a) has a 6- to 7-fold greater impact on CHD risk compared with LDL.^11,12^

Assessing CHD risk is a key aspect of any prevention strategy. It determines the nature and degree of intervention required to provide the optimum outcome for each person. LDL cholesterol (LDL-C) is used widely as an indicator of risk and as a goal to judge therapeutic success, while plasma total apoB is recognized increasingly as a more comprehensive biomarker of risk.^13^ Looking to the future, as we incorporate TRL/remnants and Lp(a) into risk assessment and into treatment guidelines, there is a need to formulate, if possible, a simple clinically useful metric(s) that provides an integrated view of an individual’s lipid-associated CHD risk. In this regard, non-HDL cholesterol (non-HDL-C) and plasma apoB have been proposed as measures of, respectively, all of the cholesterol in atherogenic lipoproteins and the total number of atherogenic particles (since each lipoprotein contains one apoB protein).^13–18^ There are scientific and practical arguments in support of each. However, these measures do not take into account differing particle atherogenicities. Algorithms could be developed that consider apoB, LDL-C, plasma triglyceride/TRL cholesterol, and Lp(a) separately, but this approach is likely to be unwieldy and complex to use in everyday practice.

In this report, we explore the benefits of generating and deploying a single metric of CHD risk based on apoB. Plasma apoB level is taken as the starting point since it represents the total number of atherogenic particles in the circulation. The proportion of apoB in TRL, Lp(a), and LDL is then estimated, and each component is weighted according to its relative atherogenicity. The resultant ‘risk-weighted apoB’ (RW-apoB), which has been explored in part previously,^19^ exhibits improved predictive power and therefore has potential clinical utility as an integrated biomarker of CHD risk.

## Methods

### Study populations

The UK Biobank cohort comprises over 502 000 UK residents of predominantly white ancestry who had the required biochemical data available.^20^ The main analyses were conducted on 285 060 subjects who were not on lipid-lowering therapy (LLT) at the time of recruitment and who had complete data with regard to LDL-C, non-HDL-C, plasma TG, apoB, and Lp(a). Further analyses were conducted on 56 442 subjects on treatment (mostly statins) (see Supplementary data online, *Figure S1*), also with the full lipid profile recorded.

The Multi-Ethnic Study of Atherosclerosis (MESA) is a prospective observational study that enrolled individuals aged 45–84 years who were free of clinically diagnosed cardiovascular disease at baseline. Participants were followed for over 10 years, with data on CHD events available throughout the follow-up period. Initial examinations took place between 2000 and 2002 across six field centres in the USA. Comprehensive information about the study design, recruitment, examinations, and data collection is available on the MESA website: https://www.mesa-nhlbi.org. Here, we analysed data from 4450 individuals with TG, Lp(a), and apoB measurements who reported not being on LLT and who had apoB levels >50 mg/dL (MESA subjects with low apoB (<50 mg/dL) discordant with their lipid profile were not included).

The Framingham Offspring Study (FOS), a prospective, community-based epidemiological survey, enrolled men and women who were either the biological children or spouses of the children of the original Framingham Heart Study cohort. Participants underwent serial examinations approximately every 4–8 years, including detailed assessments of cardiovascular risk factors, medical history, and laboratory measurements. Here, we used 2359 study participants with complete data on apoB, plasma TG, and Lp(a).

SCAPIS (Swedish CArdioPulmonary bioImage Study) recruited from the Swedish population a cohort comprising 26 049 subjects not on lipid-lowering treatment and with complete data on the same measures. SCAPIS is a population-based prospective study (www.scapis.org) that, between 2013 and 2018, randomly selected men and women aged 50–64 years from across Sweden. Registration was undertaken at six sites (Gothenburg, Linköping, Malmö/Lund, Stockholm, Umeå, and Uppsala) and subjects were invited to undergo a comprehensive health examination, as previously described.^21^ CHD events were gathered from national registries.

### Ethical review

The UK Biobank has received generic approval for data exploration of the type undertaken here. MESA and FOS received ethical approval from their respective institutional review boards (at all sites), and all participants provided written informed consent. The present analysis of the MESA and FOS data was also approved by the ethics committee of Lyon-Villeurbanne hospital, France. SCAPIS was approved by the ethics committee in Umeå, Sweden (number 2010-228-31M).

### Lipid and lipoprotein measurements

In the UK Biobank, LDL-C was measured directly (Beckman Coulter, Brea, CA). Non-HDL-C was calculated as the difference between plasma cholesterol and HDL cholesterol.^20,22^ TRL/remnant-C was derived by subtracting direct LDL-C from non-HDL-C.^5^ Lipoprotein(a) mass concentration was measured by immunoturbidimetry using the Randox assay (Data Field 30790). The assay range was 3.8–189 nmol/L (values outside the working range were classified as ‘not available’). Other analytes, including plasma apoB, were measured by standard laboratory methods. Blood samples were not obtained in the fasting state, so postprandial lipoproteins were possibly present.

In the MESA cohort, subjects were sampled after an overnight fast. Plasma lipids were measured using standard laboratory methods. apoB results were taken from the data field apobd1. Lp(a) was reported in nmol/L. In FOS, blood samples were obtained from subjects after at least a 12 h fast. Lp(a) was reported in mg/dL. In SCAPIS, subjects were fasted for at least 8 h, and Lp(a) was reported in mg/dL. Where Lp(a) was reported in mg/dL, we used a conversion factor of ×2.2 to convert the result to nmol/L.

Note that the terms ‘TRL/remnant’ and ‘TRL/remnant-C’ are used throughout the text to recognize the fact that there is no clear definition of remnant particles that allows them to be identified separately from other TRL; they are part of a continuum. The concept and nature of a TRL remnant particle are discussed in detail in the EAS Consensus Statement.^5^

### Calculation of risk-weighted apoB

RW-apoB was calculated by first estimating how much of the plasma total apoB was in TRL particles and in Lp(a). ApoB in TRL (in nmol/L) was calculated from plasma TG (expressed in mmol/L) by assuming 1 mmol/L (1 000 000 nmol/L) of plasma TG corresponds to 85 nmol/L of TRL particles (that is on average a TRL particle contains 12 000 TG molecules;^23^ while the TG/apoB ratio in VLDL does vary to a degree as a function of particle size,^24^ use of a mean value has the merit of simplicity and provided a reasonable estimate for VLDL-apoB in the calculation of RW-apoB across a wide range of plasma TG levels—see Supplementary data online, *Section S1*). Each 1 nmol/L of TRL particles contains 1 nmol/L of apoB protein. The concentration of apoB in Lp(a) was derived directly from the Lp(a) test result since each nmol of Lp(a) contains 1 nmol of apoB. For each subject, we determined TRL-apoB, Lp(a)-apoB, and by subtraction from total plasma apoB (total apoB expressed in nmol/L is the sum of all three apoB-containing lipoproteins), LDL-apoB concentrations in nmol/L. This gives particle concentrations for each lipoprotein class since 1 nmol of apoB = 1 nmol of particles. To apply a risk weighting, we took into account the previously determined relative atherogenicity of these three apoB-containing lipoprotein particles.^9,11,25^ Thus, TRL-apoB in nmol/L was multiplied by a factor of 4.5, Lp(a)-apoB in nmol/L by a factor of 6.5, and LDL-apoB in nmol/L by a factor of 1 (see Supplementary data online, *Section S1* for more detail). RW-apoB was calculated as the sum of these individual weighted apoB values. The value in nmol/L was then converted back to mg/dL by multiplying by the molecular weight of the apoB protein (530 000 Daltons). The multiplication factors were scaled so that the median RW-apoB in the population was approximately the same as the median measured plasma apoB, and 1 unit of RW-apoB was calibrated to be the risk equivalent of 1 unit of measured apoB. This allowed us to examine how the risk weighting altered the ranking of individuals within the cohort and compare this directly to the ranking based on measured apoB.

In summary, RW-apoB = 11.65 × TG + 0.215 × Lp(a) + 0.736 × ApoB, where TG is measured in mmol/L, Lp(a) in nmol/L, and apoB in mg/dL. Since it is derived from apoB, RW-apoB has notional units of mg/dL. Alternate forms of the equation and examples of calculations of RW-apoB are given in Supplementary data online, *Section S1*. The same equation (and multiplication factors) was used in all four cohorts (UK Biobank, MESA, FOS, and SCAPIS).

### Coronary heart disease events

In UKB, CHD events were defined as a combination of myocardial infarction and coronary revascularizations (see also Supplementary data online, *Table S1*). In MESA, CHD events were defined as myocardial infarction, resuscitated cardiac arrest, definite angina, probable angina followed by revascularization, and CHD-related death. Event information was obtained from hospital medical records, autopsy reports, and participant interviews. For out-of-hospital deaths, data were collected through interviews and questionnaires. In FOS, CHD was defined as myocardial infarction, angina, coronary revascularization, or CHD death. Events were adjudicated using medical records. For those without CHD, censoring dates were based on the latest exam or health update through 2019. In SCAPIS, CHD events were defined as the first occurrence of nonfatal myocardial infarction or death from CHD. Events were identified through linkage to the Swedish National Patient Register and Cause of Death Register. Mean follow-up periods were for UK Biobank 13.5 years, for MESA 12.0 years, for FOS 42.7 years, and for SCAPIS 7.8 years. In SCAPIS, the follow-up period was from the date of coronary imaging to the first CHD event, death, or the censor date of 30 September 2024.

### Statistics

All statistical analyses were performed using R version 4.3.0. *P*-values for between-group comparisons were calculated using the *t*-test for continuous variables and the χ^2^ test for categorical variables as the default in the *tableone* package. Significance tests were two-sided with a cut-off of .05 being considered statistically significant.

Modelling of CHD risk was performed using Cox proportional hazards models with the *coxph* function from the *survival* package. The models included apoB-containing lipoproteins (as independent causal factors) but not HDL-C (a non-causal factor) for the reasons given in Supplementary data online, *Section S2*. Since collinearity can affect estimates in Cox proportional hazards models, we also performed analyses to evaluate the relative predictive capacity of RW-apoB compared with apoB using the *compareC* package in R, which implements Harrell’s C-index to formally test for differences in predictive capacity between two correlated risk scores.

## Results

### Study population

The primary analysis was performed on the UK Biobank population^20^ using data from 285 060 subjects who had a complete lipid profile, including apoB and Lp(a) recorded and were not on LLT at baseline (see Supplementary data online, *Figure S1*). Further analyses were conducted on the 56 442 UK Biobank subjects who were on lipid-lowering treatment at the time of recruitment. Three other cohorts were used to replicate the analysis and validate the findings from the UK Biobank. These were MESA participants (*N* = 4450) not on lipid-lowering treatment, FOS participants (*N* = 2359) not on lipid-lowering treatment, and SCAPIS participants (*N* = 26 049) again not on lipid treatment.

### Derivation of risk-weighted apoB

**Figure 1:**
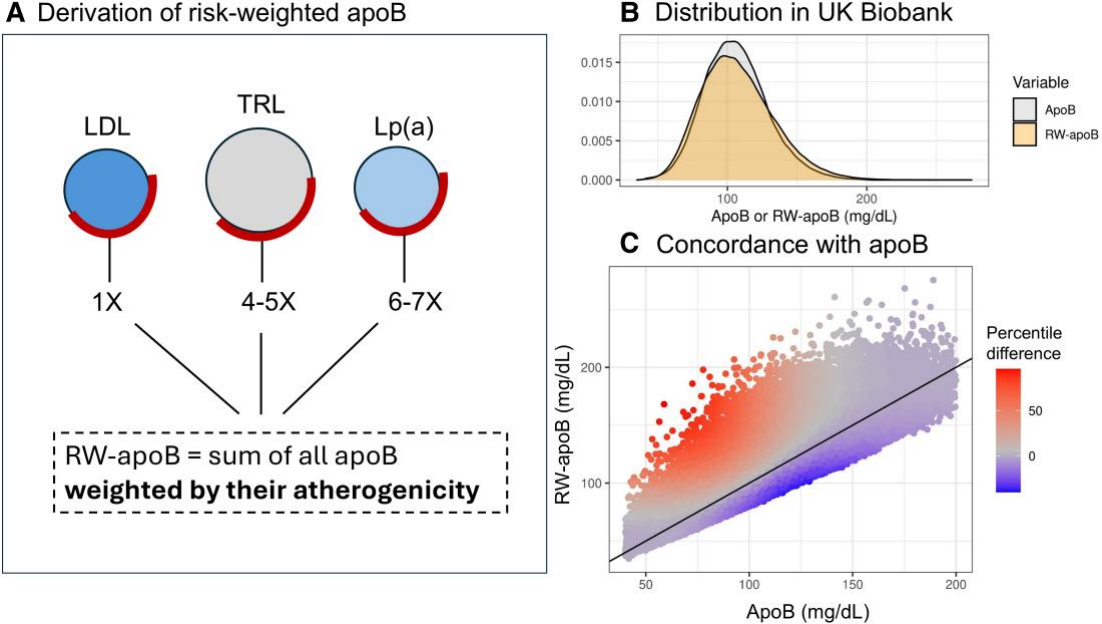
Derivation and population distribution of risk-weighted apoB. (*A*) Schematic diagram of RW-apoB derivation. The number of TRL, Lp(a), and LDL particles (based on estimated apoB content) is calculated and weighted according to their relative atherogenicity. (*B*) Measured apoB and RW-apoB distribution in UK Biobank (note the weighting factors used in calculating RW-apoB were scaled so that median RW-apoB equalled median measured apoB). (*C*) RW-apoB concordance and discordance compared with measured apoB. Colour indicates the degree of discrepancy between the percentile of apoB and RW-apoB for each individual, e.g. if a person is at the 25th percentile of apoB and the 75th percentile of RW-apoB, there is a 50-percentile difference

As set out in *Figure 1A* and Methods (see also Supplementary data online, *Section S1*), RW-apoB was derived from measured plasma total apoB using a simple equation that approximated the apoB content (=particle concentrations) of TRL, Lp(a), and LDL, and then applied weighting factors based on relative particle atherogenicity according to previously published Mendelian randomization studies.^9,11,25^ The sum of these weighted apoB components—RW-apoB—integrated for each subject the impact of all three apoB-containing lipoproteins on CHD risk.

*Figure 1B* shows the distribution of RW-apoB compared with the measured apoB from which it was derived. By design, the two distributions had the same median values (105 mg/dL; the factors used in the equations to calculate RW-apoB were scaled to achieve this outcome; see Supplementary data online, *Section S1*). RW-apoB exhibited a skewness towards higher values due to the weighting factors applied to the Lp(a)- and TRL components. However, since LDL is by far the most abundant apoB-containing lipoprotein in the circulation, the change in the overall population distribution, while evident, was not large.

### Reclassification of subjects by risk-weighted apoB

The ranking of individuals in the apoB distribution was affected markedly by the calculation of their RW-apoB, as shown in *Figure 1C*. Here, ‘concordance’ by percentile (each percentile having >2800 people) was calculated on an individual basis. That is, if a subject was in the 50th percentile of measured apoB and in the 50th percentile for RW-apoB, then they would be considered exactly concordant. However, if they had high plasma TG or high Lp(a) (or both), then the weighting would move them up the distribution, possibly by a substantial degree, and they would be counted as increasingly discordant. Over half of the whole cohort (52% of subjects) had a 10th percentile or larger difference between their ranking according to measured apoB and that according to RW-apoB. Looking at the top quintile of measured apoB, 66% of individuals also fell into the top quintile of RW-apoB, while 34% moved to a lower quintile based on their RW-apoB. Similarly, comparing the top deciles, 42% of individuals were discordant between apoB and RW-apoB.

**Figure 2:**
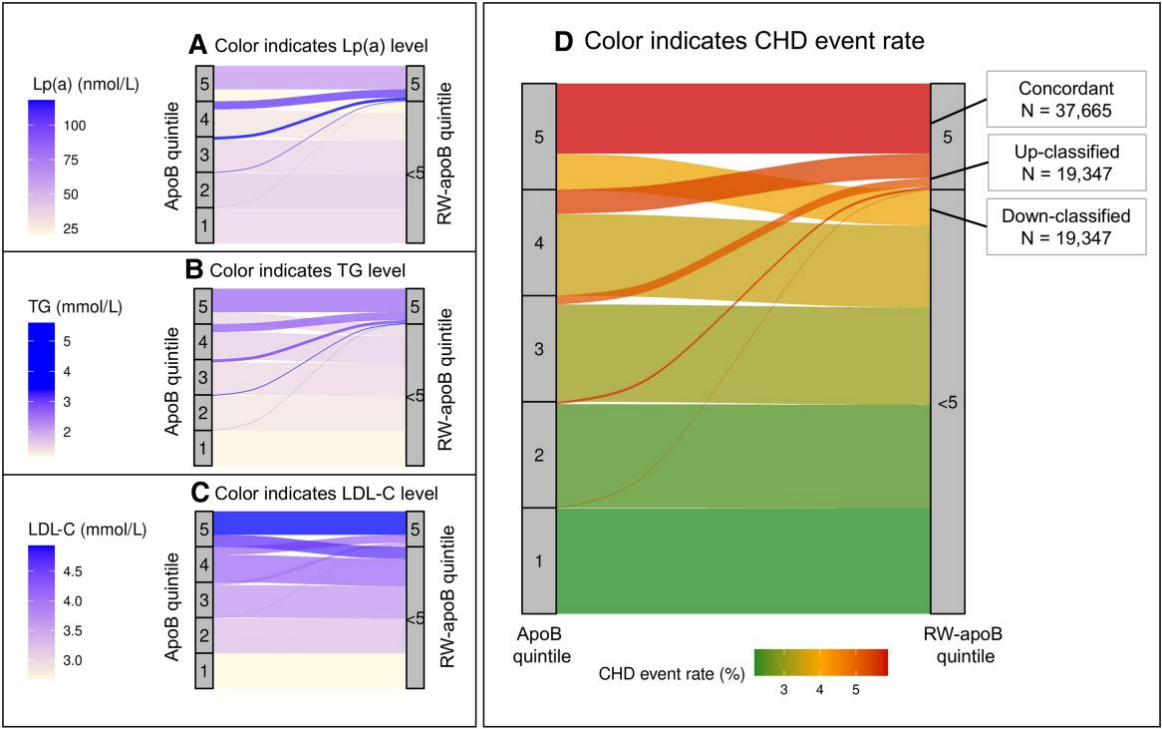
Reclassification of subjects by risk-weighted apoB. Sankey diagram with apoB quintile 1–5 in the left panels and RW-apoB quintiles 5 and 1–4 combined (i.e. <5) in the right panels, showing how individuals are reclassified (up or down) when using RW-apoB in place of apoB. 34% of individuals (*N* = 19 347) in the top apoB quintile are down-classified, and conversely, 34% of new individuals are up-classified when using RW-apoB. (*A*) Colour indicates Lp(a) levels of reclassified individuals, (*B*) colour indicates plasma TG levels, (*C*) colour indicates LDL-C levels, and (*D*) colour indicates CHD event rates of reclassified individuals. The table shows the characteristics of the individuals that are both in the top quintile of apoB and RW-apoB (concordant, *N* = 37 665) and individuals who are up- or down-classified by RW-apoB (*N* = 19 347 each). *Significant between-group difference of down- and up-classified individuals; *P* < .0001

To further assess the extent of reclassification for the whole cohort, we used Sankey diagrams that chart the flow of subjects as they are re-ranked from quintile of measured apoB to quintile of RW-apoB (*Figure 2*). For simplicity of presentation, *Figure 2A–C* depicts only subjects who were re-ranked into the top quintile (quintile 5) of RW-apoB. There were subjects in each quintile of measured apoB with high TG or high Lp(a) (an increasing proportion progressing from quintile 1 to quintile 5) who were up-classified to the top quintile of RW-apoB. Conversely, some subjects who had high measured apoB due mainly to elevated LDL moved to a lower quintile of RW-apoB (due to the lower weighting for LDL-apoB relative to TRL and Lp(a)). Supplementary data online, *Figure S2* presents Sankey diagrams for all subjects and shows the substantial reclassification by quintile that results from ranking by RW-apoB.

*Figure 2D* shows that for quintiles 1–4 of measured apoB, the subjects who were up-classified to the top quintile of RW-apoB also had a higher CHD event rate, as would be expected based on the greater atherogenicity of Lp(a) and TRL particles. The inset table in *Figure 2* gives the lipid profile of the subjects who remained in the top quintile when their RW-apoB was calculated. They had high levels of LDL-C, TG, and Lp(a). The subjects who were down-classified had high LDL-C but relatively low TG and Lp(a), while the subjects who were up-classified on the basis of RW-apoB had elevated TG and Lp(a) but near population average LDL-C. As a group, the up-classified subjects had an observed CHD event rate close to that of those who remained in the top quintile. Subjects who were down-classified on the basis of RW-apoB had a much lower CHD event rate (mean of 3.85%). Thus, after re-ranking subjects according to their RW-apoB, there was an improved gradient of CHD risk across the quintiles, with clustering of the highest risk subjects in the top quintile of RW-apoB.

### Risk-weighted apoB and coronary heart disease risk prediction

**Figure 3:**
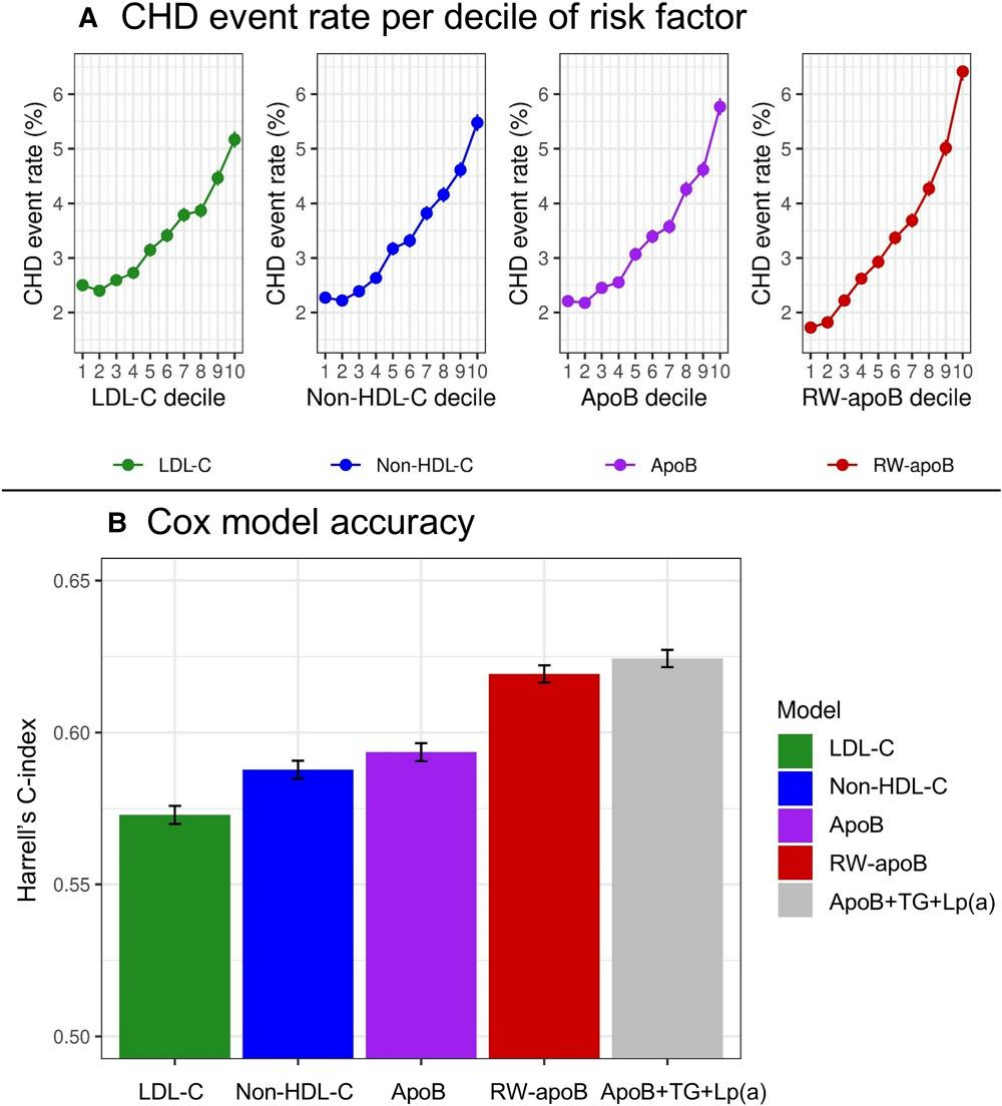
Risk prediction using risk-weighted apoB. (*A*) Coronary heart disease rate per decile of LDL-C, non-HDL-C, apoB, and RW-apoB. (*B*) Harrell’s C-index indicating the coronary heart disease prediction accuracy of each Cox proportional hazard model. Models were constructed for LDL-C, non-HDL-C, apoB, and RW-apoB separately (coloured columns). A model including apoB, Lp(a), and TG is shown in the grey column. Note: a Harrell’s C-index of 0.5 represents no predictive capacity above chance

The overall outcome of ranking subjects by RW-apoB vs measured apoB, LDL-C, and non-HDL-C is presented in *Figure 3A*. Reclassification by RW-apoB generated a steeper gradient of CHD risk across deciles as subjects with elevated TG or Lp(a) moved to higher deciles and those with primarily elevated LDL-C moved down (*Figure 2* and Supplementary data online, *Figure S2*). The gradient of CHD event rate across deciles of RW-apoB had a 3.5-fold variation from the lowest to the highest decile. Measured apoB exhibited the next strongest relationship to CHD events (lowest vs highest decile gave a 2.5-fold variation), followed by non-HDL-C (2.0-fold variation) and LDL-C (1.5-fold variation).

The calculation of Harrell’s C-index for these variables showed that RW-apoB was a significantly more accurate predictor of CHD risk compared with the other lipid predictors (*Figure 3B*). The C-index for RW-apoB was 0.619 compared with 0.591 for apoB, reflecting an absolute improvement of 0.028 (*P* < .0001). Further, RW-apoB gave a C-index result that was close to that obtained from a model that included plasma TG, Lp(a), and apoB as separate variables. Thus, RW-apoB can be used as a single metric that captures the combined risk associated with all three major apoB-containing lipoproteins; measured apoB was inferior in this regard, as shown in *Figure 3B*. This can also be seen in *Table 1*, where apoB and RW-apoB were tested in Cox proportional hazards models both in separate univariable models and together in a multivariable model. In multivariable models (i.e. mutually adjusted for each other) in both the UK Biobank and in all three validation cohorts—MESA, FOS, and SCAPIS—RW-apoB but not measured apoB remained significantly positively associated with CHD events. Since MESA included subjects from varying ethnic backgrounds, we were able to confirm in a similar multivariable model in individuals of African American descent (the second largest subgroup (*N* = 1260), other ethnic groups were too small to undertake the analysis) that RW-apoB remained a significant predictor of CHD risk while measured apoB became non-significant (data not shown).

**Table 1:** ApoB and risk-weighted apoB as predictors of coronary heart disease risk

Cohort/Model	Term	CHD Hazard ratio per 10 mg/dL	95% CI	*P*-value
UK Biobank				
Univariable	ApoB	1.144	[1.134, 1.153]	2.4 × 10^−226^
Univariable	RW-apoB	1.157	[1.150, 1.166]	<1 × 10^−300^
Multivariable	RW-apoB	1.173	[1.159, 1.187]	1.51 × 10^−154^
	ApoB	0.981	[0.967, 0.995]	.0071
MESA				
Univariable	ApoB	1.054	[1.01, 1.10]	.016
Univariable	RW-apoB	1.048	[1.03, 1.07]	7.05 × 10^−6^
Multivariable	RW-apoB	1.047	[1.02, 1.07]	2.04 × 10^−4^
	ApoB	0.961	[0.95, 1.05]	.96
FOS				
Univariable	ApoB	1.16	[1.13, 1.19]	2.85 × 10^−26^
Univariable	RW-apoB	1.19	[1.16, 1.22]	4.58 × 10^−35^
Multivariable	RW-apoB	1.19	[1.13, 1.26]	1.59 × 10^−9^
	ApoB	0.998	[0.94, 1.06]	.935
SCAPIS				
Univariable	ApoB	1.21	[1.16, 1.26]	1.4 × 10^−20^
Univariable	RW-apoB	1.17	[1.14, 1.21]	3.4 × 10^−26^
Multivariable	RW-apoB	1.13	[1.07, 1.19]	5.1 × 10^−6^
	ApoB	1.06	[0.989, 1.14]	.106

Cox proportional hazards models of apoB or RW-apoB in separate univariable models and combined in one multivariable model. Results are presented for the UK Biobank, the Multi-Ethnic Study of Atherosclerosis (MESA), the FRAMINGHAM OFFSPRING STUDY (FOS), and the Swedish CArdioPulmonary bioImage Study (SCAPIS) cohorts separately. RW-apoB, but not apoB, remains significantly positively associated with CHD events in the multivariable models. Note that the weighting factors used to calculate RW-apoB were scaled so that for the population median, RW-apoB was equal to measured apoB. Hazard ratios with 95% confidence intervals are presented.

### Use of risk-weighted apoB in clinical decision-making

We next addressed the question of how the application of RW-apoB might affect clinical decision-making in CHD prevention. In the primary prevention setting, the recommended approach is to determine a subject’s overall CHD risk and intervene according to the risk classification. In the recent ESC SCORE2 guidelines,^15^ non-HDL-C is used as the principal predictor of lipid-associated risk and as the means of classifying individuals in the population. As seen in *Figure 3A*, ranking individuals based on non-HDL-C (the range in the UK Biobank population is similar to that used in the SCORE2 tables) yields a gradient of risk that is shallower than that of RW-apoB. When RW-apoB is calculated, there is substantial reclassification of subjects (see Supplementary data online, *Figure S3*) and a marked improvement in risk discrimination (*Figure 3B*).

**Figure 4:**
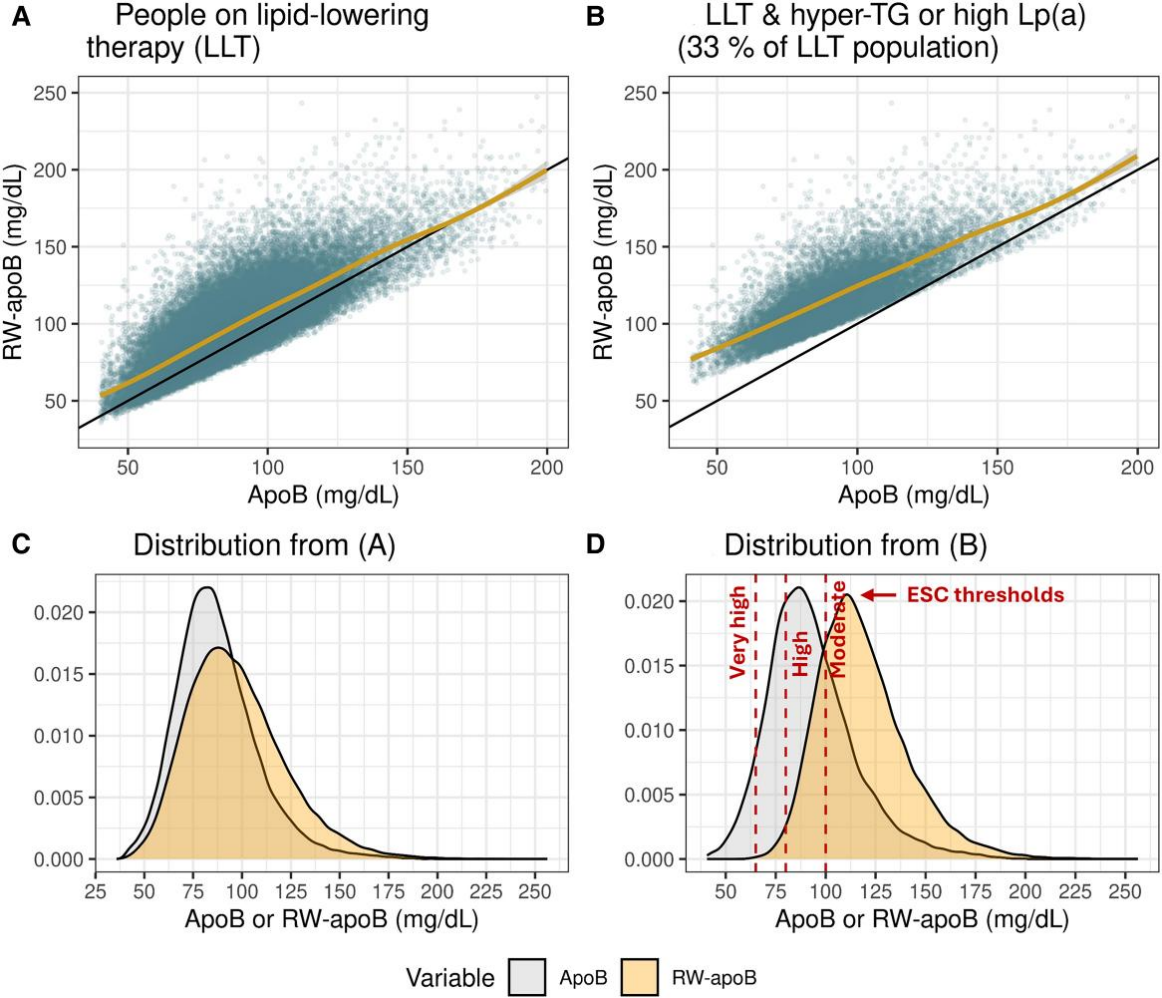
Risk-weighted apoB in subjects on lipid-lowering therapy in the UK Biobank. (*A*) Scatter plot of apoB and RW-apoB in people on lipid-lowering therapy in the UK Biobank. The black line is the line of identity, and the yellow line shows the Locally Estimated Scatterplot Smoothing (LOESS) fitted line. (*B*) Same scatter plot as in (*A*) but in UK Biobank individuals on lipid-lowering therapy with hyper-TG (plasma TG ≥ 2.5) or high Lp(a) (Lp(a) ≥125 nmol/L). (*C*) Distribution of apoB and RW-apoB from (*A*). (*D*) Distribution of apoB and RW-apoB from (*B*). Indicated are also the apoB target values in moderate-risk, high-risk, or very high-risk individuals as defined in ESC/EAS guidelines^26,27^

A further key decision point in lipid-lowering treatment strategies is whether to institute add-on therapy on top of statin treatment. RW-apoB may be of particular use in this clinical scenario. *Figure 4A* shows the distribution of RW-apoB in the 17% of the UK Biobank cohort who were on LLT at baseline (almost all were on statins). In this treated cohort, RW-apoB exhibited a more marked positively skewed distribution (*Figure 4C*). As expected, RW-apoB was markedly higher than measured apoB in subjects on LLT who had either elevated plasma TG (>2.5 mmol/L) or elevated Lp(a) (>125 nmol/L) (*Figure 4B* and *D*). Indeed, for many of these individuals, RW-apoB was 25% to 100% higher than measured apoB. Shown in the diagram are the apoB treatment thresholds recommended in the ESC guidelines.^1^ In moderate-risk individuals, the recommendation is to achieve an apoB < 100 mg/dL. If measured apoB is used as a treatment guide, then about 23% of subjects on statin will require additional treatment, but on the basis of RW-apoB, it is seen that 41% of subjects now merit further intervention. In high or very high-risk patients where the goals are apoB < 80 and <65 mg/dL, few people lie below these values for RW-apoB, and the majority will require aggressive intervention.

## Discussion

In this study, we describe the derivation, utility, and application of risk-weighted apoB, a single metric that captures the CHD risk associated with all three apoB-containing lipoproteins—LDL, TRL, and Lp(a). RW-apoB is based on measured plasma total apoB but weights the apoB estimated to be present in TRL and Lp(a) to reflect their higher per-particle atherogenicity compared with LDL. We found RW-apoB to be superior to other single predictors of CHD risk, such as LDL-C, non-HDL-C, and apoB itself. Classifying individuals using RW-apoB improved discrimination in population risk assessment schemes as advocated in international guidelines. Further, it has the potential to improve the prediction of ongoing risk in subjects in whom raised LDL has been treated with statins. The impact of using RW-apoB in patients on statin with moderate to high residual risk due to elevated TRL or Lp(a) was substantial; using RW-apoB virtually doubled the number needing further aggressive intervention and highlighted the unmet clinical need in this group. It is important to note that while RW-apoB was derived in the present report using genetically determined weighting factors for TRL, LDL, and Lp(a), upcoming intervention trials of TRL- and Lp(a)-lowering will provide further information on the strength of the association of these lipoproteins with CHD risk. This will allow further validation and refinement of the calculation of RW-apoB. Three independent validation cohorts differing in geographical location and demographic makeup were used to confirm our main finding that RW-apoB was superior in predicting CHD risk compared with measured apoB. The broader clinical utility of this metric will need to be tested in further data sets and different clinical settings, but on the basis of the present investigation, its potential as a guide in future CHD prevention strategies where LDL, TRL, and Lp(a) are taken into account is clear.

The rationale underpinning the development of an apoB-based metric that accommodates the differing atherogenicity of the apoB-lipoproteins has been explored in a previous publication that examined the derivation of an RW-apoB that took into account the atherogenicity of Lp(a).^19^ Here, the concept is expanded to include all apoB-containing lipoproteins by also incorporating TRL. Measured apoB represents the total number of atherogenic particles present in the blood circulation. Early studies suggested that all apoB-containing lipoproteins had approximately equal atherogenicity,^28,29^ but recent investigations have revealed this not to be the case. It is consistently reported that TRL cholesterol carries a higher CHD risk per mmol/L than LDL-C,^9,25,30^ and since the cholesterol to apoB ratio is higher in TRL than LDL, then the CHD risk per unit apoB must be considerably greater for TRL than LDL. To obtain an estimate of relative atherogenicity for TRL vs LDL, we identified genetic variants that affected these particles to differing degrees. Sets of variants generating a higher apoB due to increased TRL were associated with a greater CHD event rate than sets of variants that increased apoB to the same extent, but this time in LDL particles. It was estimated that TRL particles (i.e. per apoB) were about four to five times more atherogenic than LDL. Using a similar approach, we reported that Lp(a) particles were approximately 6.5 times more atherogenic than LDL. These relative atherogenicities were confirmed independently by Morze *et al.*^10^ by assessing the CHD risk associated with VLDL vs LDL particles (based on NMR measurements), and by Marston *et al*^12^ who examined the increment in CHD per unit change in apoB in Lp(a) compared with apoB in VLDL/LDL. The basis of the differential atherogenicity of TRL and Lp(a) relative to LDL is as yet unclear but is likely linked to the finding that both particles generate pro-inflammatory changes that are believed to promote atherosclerosis.^23,31^ The main advantage of deriving the weighting factors used in the calculation of RW-apoB empirically from Mendelian randomization analysis is that they are unlikely to be confounded by lifestyle or other phenotypic factors.

The finding in the present report that the observed CHD event rate per unit measured apoB was higher in those with elevated plasma TG and Lp(a) compared with those with elevated LDL-C (as seen in the RW-apoB-based re-ranking process) provides further strong support from an epidemiological perspective for our previously published genetic findings.^9,11,25^ Reclassification would not have produced improved risk discrimination if all apoB-containing lipoproteins were equally atherogenic. In our earlier publication, we reported that non-HDL-C exhibited a varying quantitative relationship with CHD risk (per mmol/L) and that this variable would need to be adjusted to improve the accuracy of risk prediction.^9^ RW-apoB addresses this issue directly and in a way that allows for both TRL and Lp(a) to be included in a simple risk metric.

Since plasma lipids in the UK Biobank were measured in the non-fasting state, the weighting factor used for plasma TG was applied not only to very-low-density lipoprotein TG but also to any chylomicron TG present. (Chylomicrons appear in a wave during absorption of fat from the diet and in most people are cleared by 8–10 h after meal consumption—these particles are usually absent from blood samples taken in the fasting state unless a subject has an elevated TG). The mean difference between a subject’s fasting and non-fasting TG level is about 0.3 mmol/L. Thus, the fasting/non-fasting status will influence the estimation of apoB in TRL. The weighting factors used in the RW-apoB calculation here, therefore, apply to non-fasting conditions. If fasting plasma TG is used, then a modified factor could be employed. There has been wide discussion as to whether fasting or non-fasting TG provides the best indicator of TRL-related CHD risk. While there are benefits in adopting fasting as a standardized state in which to assess lipid-associated risk, cogent arguments have been put forward that the inclusion of chylomicrons and their remnants in a non-fasting sample gives superior prediction of CHD risk.^32–35^ It is of interest in this regard that the calculation of RW-apoB in the three validation cohorts that used fasting TG measurements gave results that were in line with those from the UK Biobank. On this basis, we assume that the present equation for estimating RW-apoB is generally applicable, although further refinement may be required depending on population characteristics.

Achieving the best possible prediction of CHD risk is key to the development of effective prevention strategies. The assessment of an individual’s overall CHD risk is critical when screening populations to identify those who require intervention but have not yet had a cardiovascular event. Equally critical is the decision to introduce more aggressive therapy in those on first-line treatment. For lipid-related risk, non-HDL-C has been advocated for screening as in SCORE2^15–18^ and is recommended as a guide to treatment intensification in those at high risk.^1^ However, as argued in a number of recent publications and as further demonstrated in the present report, plasma apoB may be a better predictor and provide a satisfactory index of risk for those in whom LDL is the predominant apoB-containing lipoprotein.^10,28,29,36–39^ For a portion of the population, the contribution from TRL/remnants as an independent risk factor will be significant^9,23,25,40^ (although this is not a universal opinion,^41^ see Supplementary data online, *Section S2* for one explanation as to why interpretations of the strength of association of TRL with risk can differ). Since plasma TG is part of the regular lipid profile, weighing for apoB in TRL could be readily achieved along the lines described here. Also, in the future, best practice will include measurement of an individual’s Lp(a) at least once (as promoted in the recent ESC Consensus statement^42^), and this then makes possible the estimation of RW-apoB routinely. The findings of the present study support such a strategy. Use of this single metric allows an integrated assessment of CHD risk that would otherwise be difficult to achieve.

A strength of this study lies in the range of populations used as primary and validation cohorts. These include Northern European subjects of mainly white ancestry (UK Biobank, SCAPIS) and also people of other demographic and ethnic backgrounds (MESA, FOS). Use of RW-apoB needs to be refined and validated in different ethnic groups to assess its value as a globally relevant indicator of CHD risk. The weighting factors used in the derivation of RW-apoB will likely vary between ethnic groups, and the equation may need to be tailored for each. Ideally, the weighting factors would again be derived from Mendelian randomization analysis specific to each ethnic group since, as explained above, we believe that this empirical approach, independent in methodology from predictive modelling, is the best means of obtaining reliable and unconfounded estimates for these factors. Key limitations include the fact that we did not have directly measured apoB levels in the lipoprotein fractions, i.e. TRL, Lp(a), and LDL-apoB. These had to be estimated from known compositional features of the lipoprotein particles. However, such assays are not available routinely, nor are they likely to be. The fact that RW-apoB can be derived from apoB, plasma TG, and Lp(a) as measured by regular clinical chemistry assays is a considerable strength of the study and aids the general application of our findings. Additional limitations are that in the UK Biobank population, due to the performance characteristics of the assay, the Lp(a) range was truncated; values above 189 nmol/L or below 3.8 nmol/L were recorded as not available. Further, as noted in Methods, UK Biobank participants were not sampled in the fasting state. Lp(a) is not affected by fasting. These technical issues did not apply in the validation studies.

RW-apoB, based on the results in this study, can be applied readily to risk stratification to inform decisions regarding CHD prevention in individuals. While the findings in the UK Biobank in statin-treated subjects showed promise regarding the utility of RW-apoB as an index of residual lipid-associated risk, a more rigorous analysis is required in the on-treatment situation to ascertain whether the weighting factors derived in the general population are optimum for those on LLT. Testing of the predictive potential of RW-apoB in completed lipid-lowering trials will help determine its usefulness and aid in the refinement of treatment thresholds and goals. A further consideration relating to the use of RW-apoB in statin-treated subjects is the fact that we still await clinical trial evidence that lowering Lp(a) or lowering TRL/remnant levels will produce a reduction in CHD risk. Although we now have evidence from multiple sources that TRL/remnants and Lp(a) are more atherogenic on a per-particle basis than LDL, we lack data regarding the size of the benefit in lowering these lipoprotein species. If the risk reductions are modest, then emphasis will continue to be on reducing apoB principally through LDL lowering. The outcome of TRL- and Lp(a)-lowering trials will provide further information on the strength of the association between these lipoprotein species and CHD risk, permit refinement of the calculation of RW-apoB, and potentially expand its use beyond risk assessment to inclusion as a metric of treatment success.

In conclusion, this study provides evidence of the utility of a single metric that represents not only the number of apoB-containing lipoproteins in the circulation but also their atherogenicity. Likely, clinical practice in the future will routinely entail the measurement of apoB and Lp(a) as well as the traditional plasma lipid profile. RW-apoB offers a simply derived means of assessing lipoprotein-related CHD risk and has the potential to be a useful tool in clinical decision-making.

## Supplementary Material

ehaf1124_Supplementary_Data

## Declarations

### Data Availability

Data are available upon approved application to the respective organizations responsible for the clinical studies and databases utilized in this research.
