# Supplementary material for: Risk-weighted apoB: a novel summary metric outperforming traditional lipid biomarkers in predicting coronary heart disease
Source: Eur Heart J. 2026 Jan 22;47(26):3415–25. doi: 10.1093/eurheartj/ehaf1124 (PMC13337224; doi:10.1093/eurheartj/ehaf1124)
Supplement: ehaf1124_Supplementary_Data [file ehaf1124_supplementary_data.pdf]

SUPPLEMENTAL FILE

Risk-weighted apoB – a summary metric that outperforms LDL-C, non-HDL-C and apoB as a marker for coronary heart disease risk

Brief title: *RW apoB: a superior marker of CHD risk*

Michaela B Rehman[1]*, Elias Björnson[2]*, Martin Adiels[2], Jakub Morze[2,3], Göran Bergström[2], Anders Gummesson[2,4], David Erlinge[5], Tove Fall[6], Ljubica Matic[7], Stefan Söderberg[8], Carl Johan Östgren[9,10], Chris J Packard[11#] and Jan Borén[2#]

Joint first and last[#] authorship*

[1]Cardiology Department, Ramsay Santé, Médipôle Lyon-Villeurbanne, Villeurbanne, France; [2]Institute of Medicine, University of Gothenburg, Gothenburg, Sweden; [3]SciLifeLab, Department of Life Sciences, Chalmers University of Technology, Gothenburg, Sweden; [4]Region Västra Götaland, Sahlgrenska University Hospital, Department of Clinical Genetics and Genomics, Gothenburg, Sweden; [5]Department of Cardiology, Clinical Sciences, Lund University, Lund, Sweden; [6]Department of Medical Sciences, Molecular Epidemiology, Uppsala University, Uppsala, Sweden; [7]Department of Molecular Medicine and Surgery, Karolinska Institute, Stockholm, Sweden; [8]Department of Public Health and Clinical Medicine, Umeå University, Umeå, Sweden; [9]Center for Medical Image Science and Visualization (CMIV), Linköping University, Linköping, Sweden; [10]Department of Health, Medicine and Caring Sciences, Linköping University, Linköping, Sweden; [11]Institute of Cardiovascular and Medical Sciences, University of Glasgow, Glasgow, UK.

Online Table 1. Additional details on definition of CHD outcomes in UK Biobank.

**UK Biobank**		
**CHD outcome**		**Individuals, n=487,202**
Non-fatal myocardial infarction (MI)	ICD 9 codes 410, 4110, 412, 42979<br>ICD 10 codes I21, I22, I23, I241, I252	Prevalent events n = 6,577<br>Incident events n = 17,356
Fatal MI	ICD 10 codes I21, I23, I241, I251, 1252, I253, I255-I259	Incident events n = 3,850
Coronary revascularisation	**Operational procedures**<br>Codes K501, K40-K44	Prevalent events n = 2,845<br>Incident events n = 3,571
Unique CHD outcomes	First event of above	Prevalent events n = 8,391<br>Incident events n = 20,792<br>Total events = 29,183

Online Section 1. Derivation of RW-apoB and worked examples

Rationale

1. ApoB measured in plasma is the sum of the concentrations of this protein in the 3 major lipoprotein classes – LDL, triglyceride-rich lipoproteins (TRL - VLDL and chylomicrons and their remnants), and lipoprotein (a) (Lp(a)).
2. Evidence from genetic studies shows that TRL and Lp(a) have greater atherogenicity per-particle (per apoB – each particle has 1 apoB protein) than LDL. So, an apoB concentration of 100mg/dL in someone with a high Lp(a) or high plasma TG is associated with a greater CHD risk than a 100mg/dL apoB in a person where almost all of the apoB is in LDL.
3. In the absence of assays to measure TRL-apoB and Lp(a)-apoB directly, we estimated TRL-apoB from plasma TG using a factor based on the average apoB/TG ratio in TRL. Lp(a)-apoB was estimated from the measured Lp(a) concentration since 1 nmol/L Lp(a) contains 1 nmol/L apoB. LDL-apoB was obtained by subtracting TRL-apoB plus Lp(a)-apoB from total apoB.
4. CHD risk per total apoB is defined by observation in the population data set (e.g. UK Biobank) but the attribution of this risk across the 3 apoB-containing lipoprotein classes requires correction for their relative atherogenicity.
5. This attribution is achieved by calculating for each subject total apoB-associated risk = LDL-apoB x 1 +TRL-apoB x 4.5 + Lp(a)-apoB x 6.5. The coefficients used in the equations given below incorporate this calculation but are also scaled so that for the population as a whole 1 unit of RW-apoB gives the same CHD risk as 1 unit of measured apoB.
6. Calculation of RW-apoB in subjects with high TG or Lp(a) results in a value that is greater than the measured plasma apoB, while in some subjects with low TG and Lp(a) the result is less than measured apoB.
7. Re-classifying people according to their RW-apoB improves their risk assessment.

Calculation of RW-apoB from plasma lipid profile.

Equation version 1:
RW-apoB = 11.65 × TG + 0.215 × Lp(a) + 0.736 × ApoB,
where TG is measured in mmol/L, Lp(a) in nmol/L and apoB in mg/dL
Equation version 2:
RW-apoB = 0.132 × TG + 0.215 × Lp(a) + 0.736 × ApoB,
where TG is measured in mg/dL, Lp(a) in nmol/L and apoB in mg/dL.
Equation version 3:
RW-apoB = 0.132 × TG + 0.473 × Lp(a) + 0.736 × ApoB,
where TG is measured in mg/dL, Lp(a) in mg/dL and apoB in mg/dL.

Worked examples:

Subject 1 lipid profile: TG 1.4 mmol/L, Lp(a) 10 nmol/l, apoB 80 mg/dL
RW-apoB = 11.65 × 1.4 + 0.215 × 10 + 0.736 × 80 = 77.3

Subject 2 lipid profile: TG 3.2 mmol/L, Lp(a) 10 nmol/l, apoB 110 mg/dL
RW-apoB = 11.65 × 3.2 + 0.215 × 10 + 0.736 × 110 = 120.4

Subject 3 lipid profile: TG 2.0 mmol/L, Lp(a) 170 nmol/l, apoB 70 mg/dL (on statin)
RW-apoB = 11.65 × 1.4 + 0.215 × 10 + 0.736 × 80 = 111.4

Estimation of VLDL-apoB from plasma triglyceride concentration.

As described in Methods, we used a fixed, average ratio to calculate VLDL-apoB from plasma TG. Based on published compositional data it was estimated that 1 mmol/L (1,000,000 nmol/L) of plasma TG corresponded to 85 nmol/L of TRL particles (that is on average a TRL particle contains 12,000 TG molecules). Since each VLDL particle has a single apoB protein VLDL-apoB was taken as 85 nmol/L per 1.0 mmol/L of plasma TG.

It is known that there is a degree of variation in the apoB content per VLDL particle with larger particles having a higher TG/apoB ratio than smaller particles. However, even at elevated TG the number of smaller VLDL still far exceeds the number of large VLDL. We used the recent data published by Cole et al[1] on measured VLDL-apoB concentration as a function of increasing plasma TG levels to model the effects of varying the VLDL-apoB/plasma TG ratio.

In the plot below RW-apoB calculated with a fixed value for the VLDL-apoB to plasma TG ratio (x-axis) was compared to RW-apoB calculated using a variable VLDL-apoB to plasma TG ratio based on the data of Cole et al[1] (y-axis). The correlation between the two RW-apoB estimates was very high at r^2=0.98. We concluded that use of a fixed, average VLDL-apoB/plasma TG ratio gave acceptable results, and provided a simple means of calculating RW-apoB with an appropriate accuracy.

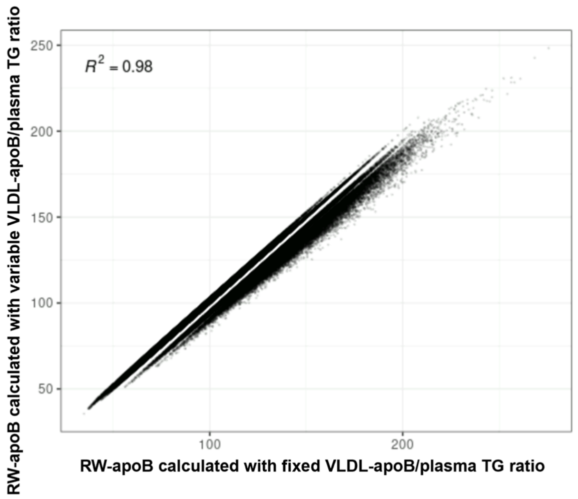

Figure: Scatterplot of RW-apoB calculated using a fixed ratio of VLDL-apoB/plasma TG vs a variable ratio based on data from Cole et al which covered the plasma TG range 0.85-4.25 mmol/L.[1]

Online Section 2. Inclusion or non-inclusion of HDL-C in predictive statistical models affects estimated strength of TRL association with risk.

In predictive models using TRL as exposure, it is important to appreciate that the strength of the association with CHD risk can be diminished significantly if HDL-C is included as a covariate. We do not include HDL-C in the models used in this paper based on the following rationale.

TRL are considered independent risk factors for CHD. This is based on a series of genetic/ Mendelian randomisation studies that document the causal association of factors that affect TRL with altered risk of CHD. HDL, in contrast, is considered as not causally linked to CHD. Genetic studies fail to show a causal association, and HDL raising intervention trials with nicotinic acid and CETP inhibitors report no benefit from increasing HDL.

HDL is linked metabolically with TRL. When TRL are elevated, there is transfer of cholesteryl ester from HDL in exchange for triglyceride. TG enriched HDL particles are more open to hepatic lipase mediated lipolysis and the action of this enzyme reduces the core TG in HDL and decreases the size of the particle. ApoA1 the main HDL protein has a reduced affinity for HDL particles as they become smaller due to a change in conformation, and as a result there is desorption of apoA1 into plasma and this free apoA1 is cleared rapidly. Kinetic studies reveal consistently a strong positive relationship of plasma TG to apoA1 clearance rates. The loss of apoA1 reduces the number of HDL particles in the bloodstream. Thus, elevated TG causes low HDL through this well-known metabolic relationship. Further, HDL-C is affected by both VLDL and chylomicrons by the same CETP/HL mediated mechanism. Measurement of fasting TG or early morning TG may not reveal the full impact of TRL on HDL-C, and it is reasonable to accept that a low HDL-C is a measure of the integrated effect of elevated VLDL and the multiple waves of chylomicrons that are secreted from the gut throughout the day.

The cause-effect relationship of TRL with HDL is demonstrated clearly in clinical trials of apoCIII inhibition. Blocking apoCIII action lowers TRL concentrations markedly and generates a substantial rise in HDL-C and HDL particle number [2].

In a predictive statistical model, it is important to include causal factors that are independent of each other (TRL, LDL-C, Lp(a)) but not confounding, non-causal factors that are related strongly to, and therefore potentially dilute the relationship of, any of these lipoproteins to CHD risk. Arguably, if HDL-C was causal then it should be included in the model, if it is demonstrably non-causal, it will diminish the association of any causal factor with which it is statistically associated, namely TRL.

Thus, in the predictive models that we used in the present paper, we do not include HDL-C for the reasons rehearsed above. Some investigators have included HDL-C in assessing the relationship of TRL to CHD risk and report much weaker associations with CHD as a result [3]. This difference in approach generates different views of the overall contribution of TRL to CHD risk.

Online Figure 1

UK Biobank flowchart of subject selection

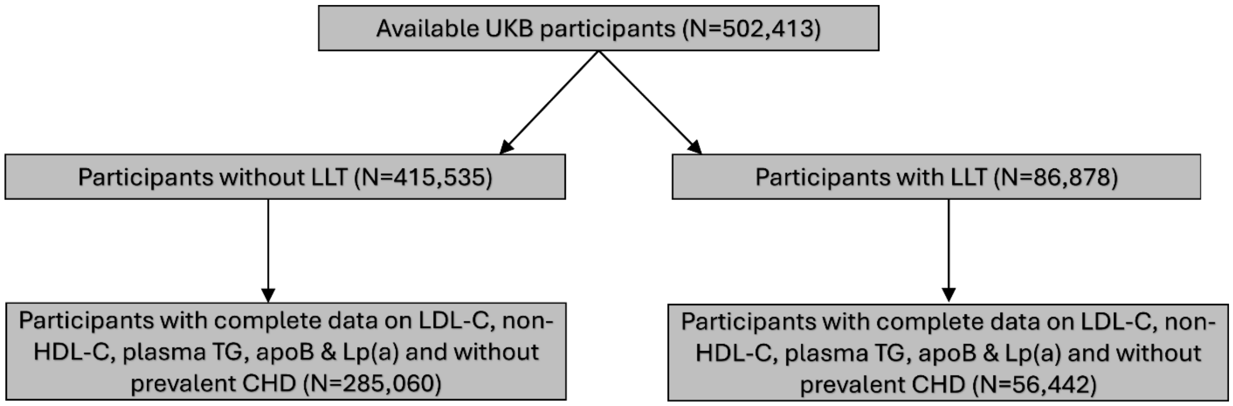

LLT = lipid lowering therapy

Online Figure 2

Sankey diagrams showing subject reclassification from measured apoB to risk-weighted apoB by quintile. Colour indicates plasma concentrations of A) Lp(a), B) plasma TG and C) LDL-C. Individuals with high Lp(a) and TG tend to be up-classified by RW-apoB and individuals with isolated elevated LDL-C (but low Lp(a) and plasma TG) tend to be down-classified.

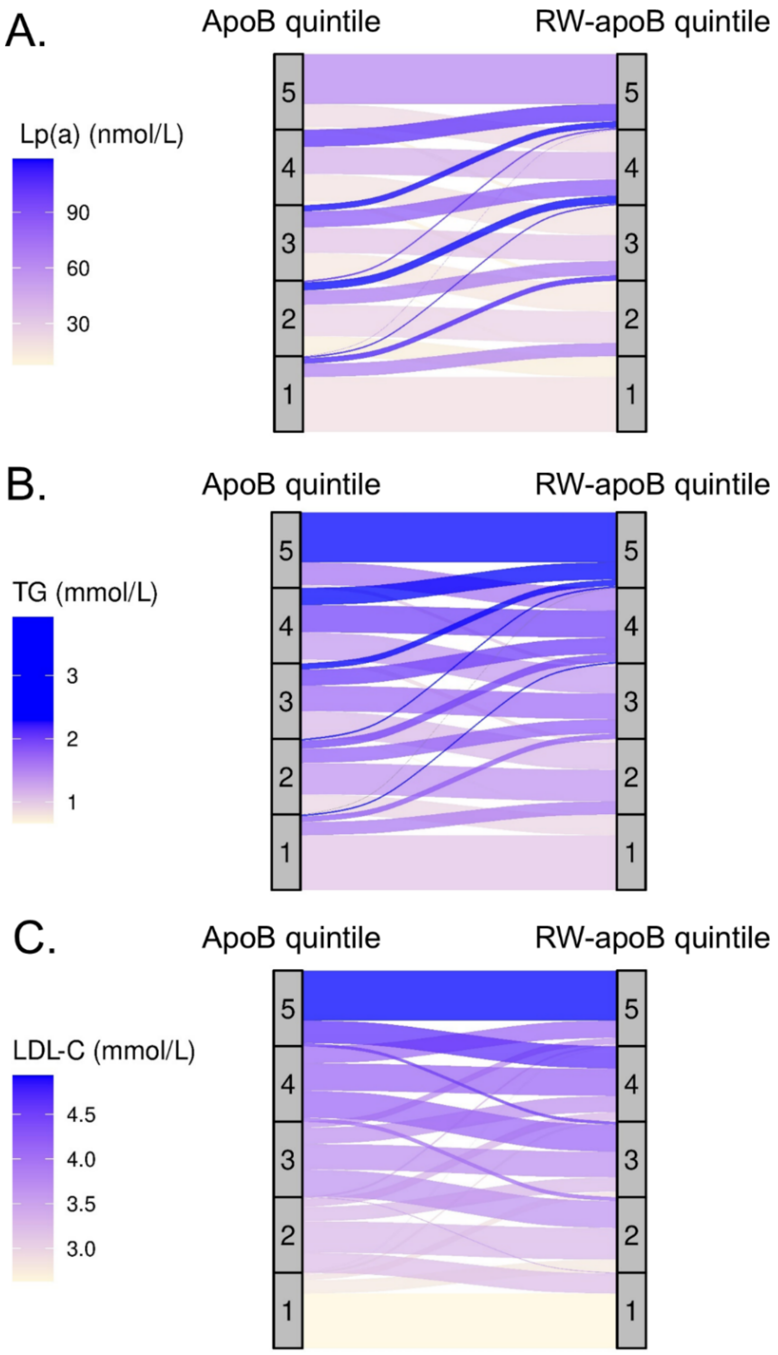

Online Figure 3

Sankey diagram showing subject reclassification from measured non-HDL-C to risk-weighted apoB by quintile. Colour indicates CHD event rate. People with high CHD risk are present in lower quintiles of non-HDL-C and they tend to be up-classified by RW-apoB.

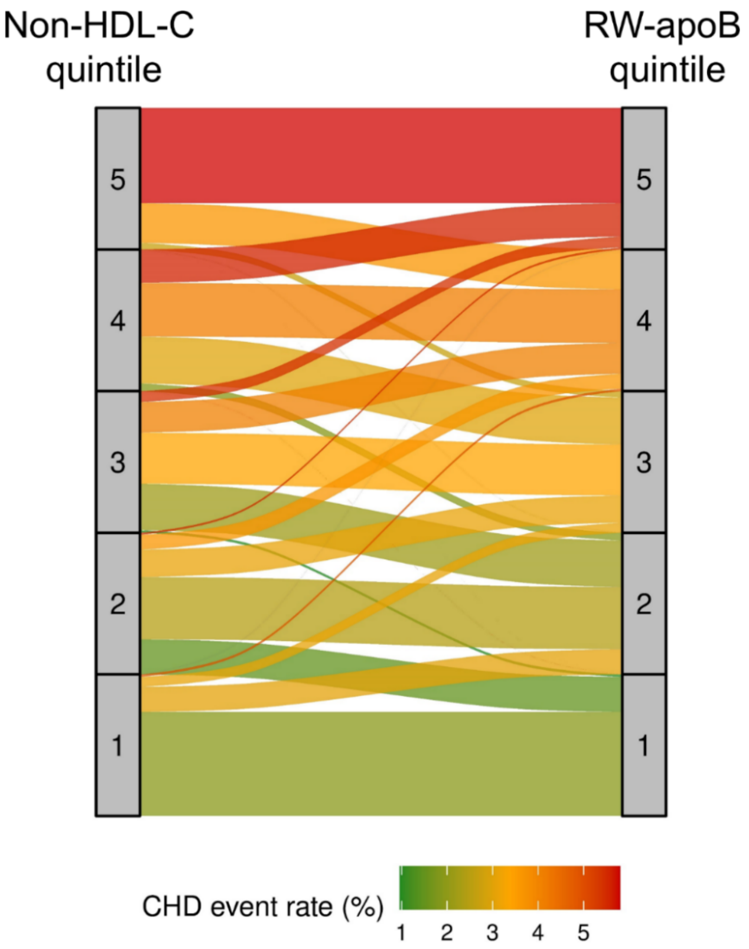

REFERENCES

1. Cole J, Couture P, Tremblay AJ, Sniderman AD. Variance in the composition and number of VLDL and LDL particles with increasing triglyceride or increasing ApoB concentrations. J Clin Lipidol 2025;**19**(1):72-82.

2. Ballantyne CM, Gaudet D, Rosenson RS, Hegele RA, Zhou R, Melquist S, *et al.* Effect of Targeting ApoC-III With Plozasiran on Lipoprotein Particle Size and Number in Hypertriglyceridemia. J Am Coll Cardiol 2025;**85**(19):1839-1854.

3. Bilgic S, Pencina KM, Pencina MJ, Cole J, Dufresne L, Thanassoulis G, *et al.* Discordance Analysis of VLDL-C and ApoB in UK Biobank and Framingham Study: A Prospective Observational Study. Arterioscler Thromb Vasc Biol 2024;**44**(10):2244-2251.
